# Rational Choice or Altruism Factor: Determinants of Residents’ Behavior toward Household Waste Separation in Xi’an, China

**DOI:** 10.3390/ijerph191911887

**Published:** 2022-09-20

**Authors:** Zhaoyun Yin, Jing Ma

**Affiliations:** School of Public Policy and Administration, Xi’an Jiaotong University, Xi’an 710049, China

**Keywords:** rational choice, altruism factor, household waste separation, behavior

## Abstract

Understanding why people do or do not perform household waste separation is a vital premise for designing relevant policies to promote waste management. As such, in this paper, an empirical study was carried out to explore the impacts of both rational choice and altruism factors on residents’ household waste separation behavior. Through the analysis of the survey sample (*n* = 1102) from Xi’an, China, using structural equation modeling, the main findings suggested that (i) the rational choice model can better explain such behavior, (ii) the altruism factor cannot directly affect household waste separation behavior, (iii) the altruism factor is highly correlated with the attitude determinant of household waste separation behavior, and (iv) rational choice models incorporating the altruism factor may have better explanatory efficacy. After that, some factors influencing residents’ altruism to household waste separation were identified. The main aim of this study was to compare two different tendencies in explaining sustainable behavior and help to find a better framework for behavior analysis.

## 1. Introduction

At present, waste management has emerged as one of the most basic tasks for the government [1]. Unfortunately, the World Bank claimed that more than 3 billion tons of municipal solid waste are generated globally every year [2]. A large amount of waste is not only harmful to human health but also poses a serious challenge to the natural ecosystem’s capacity [3]. Therefore, inadequate waste management would compromise the sustainable development of our society.

Motivated by the concept of sustainable development, the traditional way of using simple disposal in waste management should be abandoned. An advanced waste management system has been designed to include six operations, i.e., waste generation, household waste separation, selective collection, transport, processing and transformation, and disposal [4]. There is no doubt that as the front end of this management chain, household waste separation plays a vital role in determining both the quantity and quality of waste flowing during the follow-up processing procedures. In other words, household waste separation is the most crucial step in the success of waste management [5]. However, excluding several developed countries (e.g., Germany and Japan), the deployment of selective collection is still limited in many nations, particularly in developing countries [6]. Thus, from a theoretical and substantive point of view, effective measures must be taken to promote this action.

In recent decades, researchers have found that the best prediction of behavior is obtained by asking people if they intend to behave in a certain way (e.g., the Theory of Planned Behavior) [7,8,9]. Obviously, these studies follow a basic presumption: household waste separation is a self-interested behavior guided by rational choice. As evidenced, many rational choice models (e.g., the Theory of Reasoned Action) are widely used in the behavioral analysis of household waste separation [5]. Further, economic factors (i.e., the important factors considered in rational choice) have been proven to impact waste recycling [10]. However, this basic presumption is facing more and more challenges. Rational choice has a limited explanatory effect because human behavior (including decision-making) is ordinarily automatic or susceptible to interference by irrational information [11]. Some researchers have stated that a complete understanding of people’s behavior should consider biological and evolutionary roots [12]. On these grounds, we inferred that the altruism factor, a manifestation within the evolutionary motivation of organisms [13], may be an important determinant of residents’ behavior toward household waste separation. Relevant research has also shown that the altruism factor can drive waste recycling [14]. To sum up, although rational choice and the altruism factor may both contribute to explaining household waste separation behavior, it is uncertain which of them really dominates the decision. The lack of differentiation and predictability hinders the development of knowledge that can better understand and promote household waste separation and public policy. Therefore, it is necessary to study which tendencies (i.e., rational choice or altruism factor, or even a combination of them) can better explain household waste separation behavior.

Aiming to comprehensively investigate the impact of rational choice and altruism factor on household waste separation behavior, this study applies a case study from Xi’an, China, a city which has a per capita GDP around the average level of the major cities in the country. Our findings can be helpful in identifying the variables and constraints in order to design policies to promote household waste separation. Furthermore, the analysis results of the altruism factor will help to determine a better framework for behavior analysis since traditional research focuses on the field of rational choice.

## 2. Theoretical Background and Hypotheses

### 2.1. Theory of Planned Behavior

**Figure 1:**
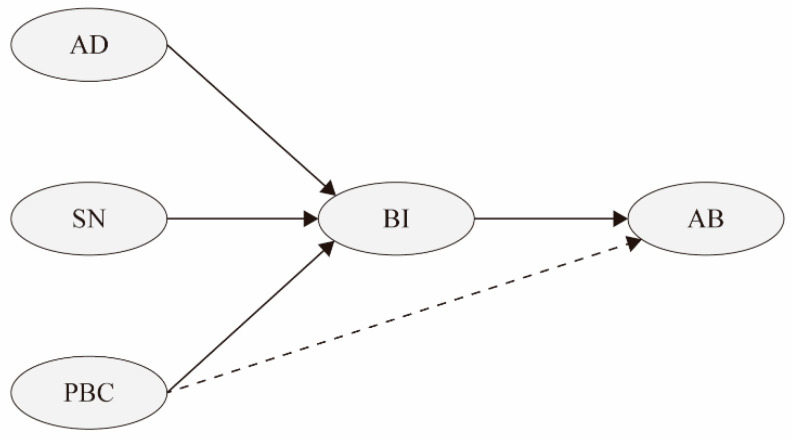
The standard TPB model. Notes: AD = attitude determinant; SN = subject norm; PBC = perceived behavioral control; BI = behavioral intention; AB = actual behavior.

As an important rational choice theory, the Theory of Planned Behavior (TPB) was developed from the Theory of Reasoned Action, which assumes that people are rational and will fully consider the significance and consequences of behavior before taking a certain action [15]. The standard TPB contains five elements (see Figure 1), i.e., attitude determinant, subjective norm, perceived behavioral control (PBC), behavioral intention, and actual behavior. In addition to these elements, TPB allows the introduction of other elements to enhance its explanatory efficacy [16]. In terms of applications, TPB is widely used to understand various behaviors, such as waste recycling [17], exercise behavior [18], and touring behavior [19]. Today, the reliability of TPB in rational analysis of behaviors is supported by more and more meta-analysis evidence [10].

Accordingly, TPB has also been adopted to explain or predict household waste separation behavior, especially in some cities in China. For example, Zhang et al. [20] investigated household waste separation in Guangzhou using an extended TPB model. In the study of Xu et al. [21], an extended TPB model with moral obligation and past behavior was used to explore residents’ waste separation behavior at the source in Hangzhou. In addition, Ma et al. [4] used another extended TPB model to analyze residents’ household waste separation behavior in Guilin. Previous studies showed that attitude determinants, subjective norms, and PBC seem to play an important role in determining residents’ waste separation intention, and behavioral intention may be the most important antecedent of waste separation behavior.

Motivated by this, this paper proposes the following hypotheses:

**H1.**  *Attitude determinants are positively related to household waste separation intention*.

**H2.**  *Subjective norms are positively related to household waste separation intention*.

**H3.**  *Perceived behavioral control is positively related to household waste separation intention*.

**H4.**  *Household waste separation intention is positively related to household waste separation behavior*.

### 2.2. Altruism Factor

The altruism factor is defined as “a form of unconditional kindness without the expectation of reciprocity” [22]. Further, the altruism factor has been variously described as “voluntary helping actions where one attempts to improve the welfare of others at some cost to oneself” [23]. Regardless of the definition, it can be distinguished that the altruism factor is significantly different from self-interested rational choice. Guagnano [24] even argued that the contrast between the two is a central theme in the social sciences. In practice, the altruism factor is regarded as the main motivation for people to take on certain behaviors (especially pro-environmental behavior). For example, evidence suggests that the altruism factor can stimulate waste recycling and green buying [14]. It was believed that any successful solution to an environmental problem requires significant sacrifices by people, forcing them to reject egoistic tendencies, thereby forming an altruistic relationship [25]. Therefore, we propose the following hypotheses:

**H5.**  *The altruism factor has a positive impact on household waste separation intention*.

**H6.**  *The altruism factor has a positive impact on household waste separation behavior*.

Different from the above viewpoint (i.e., rational choice and altruism factor are independent of each other), some scholars believe that the altruism factor could be considered an egoistic preference since it brings subjective satisfaction to the actor [26]. This means the altruism factor should be incorporated into rational choice models to refine the analysis of behavior [27,28]. Altruistic preference and rational choice preference are interdependent to some extent [26], e.g., self-sacrifice can be used as an investment to show that the actor promotes group values [29]. Thus, based on the TPB model, this paper hypothesizes that:

**H7.**  *The altruism factor has a positive impact on the attitude determinants of household waste separation behavior*.

**H8.**  *The altruism factor has a positive impact on the subjective norms of household waste separation behavior*.

**H9.**  *The altruism factor has a positive impact on the perceived behavioral control of household waste separation behavior*.

## 3. Methods

### 3.1. Study Area

**Figure 2:**
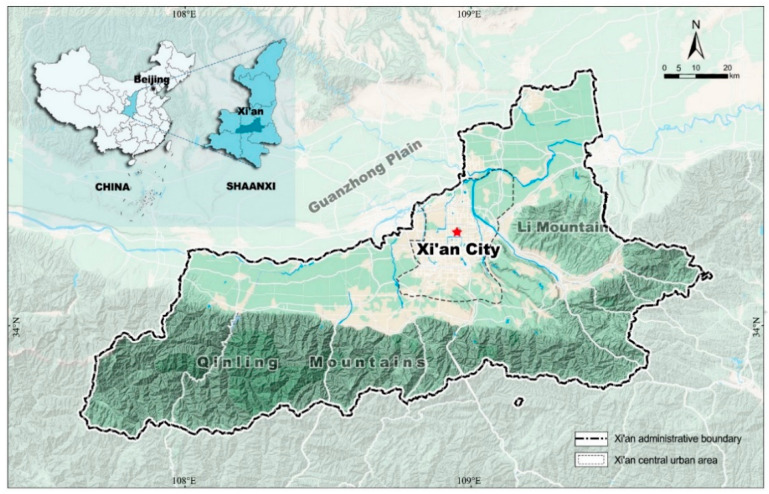
Location of Xi’an, China.

As shown in Figure 2, Xi’an is located in the mid-part of Shaanxi province of China, which has a per capita GDP around the average level of the major cities in China [30]. Its geographic location is longitude 107.40–109.49° and latitude 33.42–34.45°. More importantly, Xi’an is one of the 46 key cities identified by the Ministry of Housing and Urban-Rural Development of China (MHURD) to promote municipal solid waste selective collection [31]. In addition, as the largest developing country in the world, the experience of Chinese cities in waste management has important reference value for sustainable development in other developing nations [32,33].

Following the requirements of MHURD, in May 2019, the City Management Committee of Xi’an announced that it was introducing a policy called the “Implementation scheme of domestic waste selective collection in Xi’an in 2019” [34]. The policy included a series of measures to improve residents’ willingness toward waste selective collection, especially through cultivating good environmental values, such as altruistic values.

### 3.2. Data Source

The research data were derived from a social survey conducted in the central urban area of Xi’an (i.e., Baqiao, Beilin, Lianhu, Weiyang, Xincheng, and Yanta districts) in August 2020. The survey adopted the stratified random sampling method. Specifically, through web crawler technology, we randomly selected three communities with high, medium, and low economic levels in each district according to rental price information (information from: lianjia.com). As a result, the residents living in the 18 selected communities formed the subjects of this study. In total, 1583 residents participated in the survey; however, only 1102 responses were valid in the sample, as shown in Table 1.

### 3.3. Measurement Instrument

#### 3.3.1. TPB Measures

All items for the TPB model followed the guidelines of previous similar studies [35,36,37], including (i) the attitude determinant was measured using four items (e.g., “Household waste separation is good”), (ii) subjective norms were captured with five items (e.g., “My family expect me to separate household waste”), (iii) five items were included to capture PBC (e.g., “Whether or not I separate household waste is entirely up to me”), (iv) household waste separation intention was assessed using three items (e.g., “I plan to separate household waste”), and (v) household waste separation behavior measured by the item “I never/often separate my household waste” was selected. The response format for each measure was scored using a Likert five-point scale from “strongly disagree” to “strongly agree”. The specific questions of the survey are shown in Appendix A.

#### 3.3.2. Altruism Measures

The existing literature lacks the standard for measuring the altruism factor since scholars have disputes about its conceptual boundary [14,25]. Generally speaking, the altruism factor can be categorized into pure altruism (i.e., when a person is driven to protect the environment, even at some individual sacrifice) and competitive altruism (i.e., when a person wants to appear to be helpful but acts with the purpose to improve personal reputation) [14]. However, only pure altruism fits the interest of this study because household waste separation is related to communal reciprocity and offers little room for reputation. Further, in order to facilitate measurement, we believe that the altruism factor is a special case of consequence consciousness (i.e., altruistic consequence consciousness). Meanwhile, in Chinese traditional culture (such as Liao-Fan’s Four Lessons), being beneficial to the public is also an important embodiment of the altruism factor [38,39]. Therefore, according to the connotation of pure altruism, four items were used to measure residents’ altruism factor, e.g., “Household waste separation can provide a better environment for others (such as human offspring)”. The specific questions of the survey are shown in Appendix A.

### 3.4. Statistical Analysis

In this study, structural equation modeling was employed using AMOS 23.0 to build a measurement model and verify the relationship between variables. Structural equation modeling can integrate the factor and path analysis methods, effectively deal with the structural relationship among multiple variables, and overcome the collinearity among independent variables [40,41]. By extending traditional multivariate analysis methods (e.g., regression, factor analysis, and correlation analysis), AMOS 23.0 can provide effective support for theoretical research [42].

Moreover, a mediating effect test for correlated variables may be required during statistical analysis. Thus, Bootstrap’s mediation test, a powerful method for mediation analysis, was introduced to test whether the 95% confidence interval of the product term of the regression coefficient contained 0 [43]. If the 95% confidence interval did not contain 0, there was a mediating effect. Otherwise, there was no mediating effect.

In addition, the influencing factors of the determinants (e.g., altruism factor) need to be explored in order to better make recommendations to promote household waste separation. Therefore, regression models were used to explore the influencing factors since they are more suitable for analyzing the specific effect path between variables than other models, such as structural equation modeling [44]. The baseline regression model adopted the ordinary least squares, and the equation can be written as

Yi = α + β Xi + εi,(1)

where Yi denotes the determinants of residents’ behavior to household waste separation (e.g., altruism factor), α is the model constant, β is the regression coefficient, Xi represents the influencing factors, and εi is the error term.

## 4. Results

### 4.1. Measurement Model

**Figure 3:**
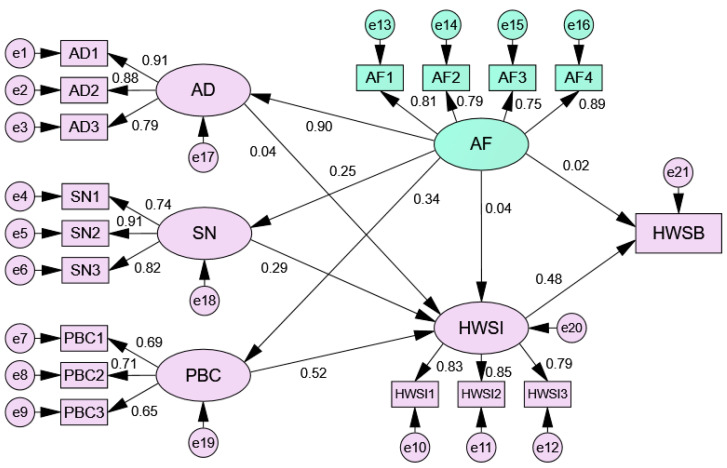
Path analysis of research model. Notes: AF = altruism factor; AD = attitude determinant; SN = subject norm; PBC = perceived behavioral control; HWSI = household waste separation intention; HWSB = household waste separation behavior; Only the items that can be used for confirmatory factor analysis were reported (factor loading > 0.6).

The measurement model evaluation follows the requirements of previous studies [45,46]. The specific model path is shown in Figure 3.

The model fit indices are reported as follows: (i) the root mean square error of approximation (RMSEA) = 0.048 < 0.08 (suggested threshold); (ii) χ^2^/df = 3.577 < 5 (suggested threshold); (iii) the root mean square residual (RMR) = 0.042 < 0.05 (suggested threshold); (iv) the Bentler comparative fit index (CFI) = 0.973 ≥ 0.9 (suggested threshold); (v) the goodness of fit index (GFI) = 0.958 ≥ 0.9 (suggested threshold); (vi) the adjusted goodness of fit index (AGFI) = 0.943 ≥ 0.9 (suggested threshold); (vii) the Bentler–Bonnett normed fit index (NFI) = 0.963 ≥ 0.9 (suggested threshold); (viii) the Tucker–Lewis non-normed fit index (NNFI) = 0.967 ≥ 0.9 (suggested threshold); (ix) the Bollen relative fit index (RFI) = 0.955 ≥ 0.9 (suggested threshold); and (x) the Bollen incremental fit index (IFI) = 0.973 ≥ 0.9 (suggested threshold). Note that when the proposed model fits the actual data perfectly, the model parameters have a unique solution, which means that the model fitting indices reach the extreme value, such as RMSEA is 0, CFI is 1, and TLI is 0. However, most models can hardly fit the actual data perfectly due to data deviation or problems in the model itself [47]. Hence, the researchers proposed the suggested thresholds of the fitting indices as the criteria to judge whether the proposed model can meet the research needs [46]. It can be seen that in this study, the fit indices were within the thresholds of the recommended values, showing that the proposed model fits the data well.

Furthermore, the average variance extracted (AVE) of the structure of the measurement model ranged from 0.468 to 0.742, the composite reliability (CR) ranged from 0.725 to 0.896, and the Cronbach’s alpha (α) ranged from 0.716 to 0.893, which clearly demonstrates that all measures have acceptable reliability [45,46].

In addition, the discriminant validity needs to be examined, as discriminant validity involves the degree to which one construct is truly different from other constructs (also used to assess the reliability of constructs). As shown in Table 2, the component scores of each construct were higher than others, which indicates the model has good discriminant validity and necessary construct validity [40]. In summary, the measurement model satisfies the rule of thumb.

### 4.2. Hypothesis Testing

As shown in Table 3, the results of path analysis show that SN (β = 0.29, *p* < 0.001) and PBC (β = 0.52, *p* < 0.001) had positive and significant effects on household waste separation intention. HWSI (β = 0.48, *p* < 0.001) had positive and significant effects on household waste separation behavior. Meanwhile, AF had positive and significant effects on AD (β = 0.90, *p* < 0.001), SN (β = 0.25, *p* < 0.001), and PBC (β = 0.34, *p* < 0.001), respectively. Thus, H2, H3, H4, H7, H8, and H9 were supported. However, AD (β = 0.04, *p* = 0.660) had no significant effects on household waste separation intention and AF had no significant effects on household waste separation intention (β = 0.04, *p* = 0.687) and household waste separation behavior (β = 0.02, *p* = 0.486). Hence, H1, H5, and H6 were not supported.

## 5. Discussion

### 5.1. Determinants of Residents’ Behavior to Household Waste Separation

In this study, two different tendencies, rational choice and the altruism factor, were included in the analysis as the determinants of household waste separation behavior. According to the research results, we found that the rational choice model can better explain such behavior. Subjective norms and PBC were the main predictors of household waste separation intention. This is consistent with many previous research conclusions [41,48]. Subjective norms refer to the influence of external social pressures on people’s specific behavior [15]. Evidence suggests that the greater the external social pressure, the stronger the individual’s willingness to participate (such as household waste separation), especially in a collectivist country such as China [41]. Therefore, H2 was supported. PBC refers to people’s perception of the difficulty in carrying out a specific behavior [15]. Generally speaking, residents inevitably face some difficulties in household waste separation. For example, without professional knowledge, it is difficult for residents to separate household waste [49]. Hence, once residents think household waste separation is not difficult, the possibility of such behavior may increase. Therefore, H3 was supported. Likewise, H4 was supported because the stronger the intention to engage in the behavior, the greater the likelihood of its performance [15]. However, in this study, we found that the attitude determinant has no effect on household waste separation intention. The understandable explanation may be that Xi’an has carried out a lot of information publicity [34], which has led the attitudes of residents toward the behavior of household waste separation to increase. Unfortunately, such an increase in attitude is difficult to translate into behavioral intention. The reason is that in China, people’s intention to separate household waste mainly results from personal tendency, including subjective norms, PBC, and economic benefits [4]. Therefore, H1 was not supported.

Furthermore, although the altruism factor can drive some sustainable behaviors (such as waste recycling and green buying) [14], it could not directly affect waste separation behavior at the source, which is an important finding of this study. However, the altruism factor was related to subjective norms and PBC. This means that the altruism factor may have an indirect impact on household waste separation behavior through these two determinants. The results passed Bootstrap’s mediating effect test (*p* < 0.001). The reasonable explanation is that the Chinese government widely publicizes the altruistic value of “all for one and one for all” in society [50]. This internalization of values effectively forms residents’ subjective norms for household waste separation. Meanwhile, the altruism factor may help overcome the difficulties encountered in performing household waste separation behavior since it is a behavioral driver [14]. In this regard, we believed that the altruism factor could promote such behavior. In addition, the altruism factor was highly correlated with the attitude determinant of household waste separation behavior (β = 0.90, *p* < 0.001). The possible reason is that the altruism factor can significantly increase an individual’s attention to environmental issues, thereby affecting an individual’s attitude determinant of household waste separation behavior [51]. Our previous study suggested that although the increase in attitude determinants may not promote sustainable behaviors (e.g., waste recycling), the decrease in attitude determinants may inhibit them [17]. Therefore, there may be more potential benefits of promoting the altruism factor. All in all, our study supported the view that the altruism factor should be incorporated into a rational model [27,28]. As shown in Table 4, the variance changed, indicating that the introduction of altruistic factors increased TPB’s explanation of household separation intention and behavior by 4% and 1%, respectively [52].

### 5.2. Influencing Factors of Altruism Factor

Although many articles have been published to explore the influencing factors of TPB, less research analyzed these factors as altruism factors. Thus, in this paper, our study empirically analyzed the influencing factors of the altruism factor. First, the demographic variables were taken as the independent variables of the baseline model, and then other potential explanatory variables, such as policy, information intervention, and knowledge of household waste separation, were added to the baseline model. The results of multiple regression are shown in Table 5.

Age (β1 = 0.028, *p* < 0.01; β2 = 0.014, *p* < 0.05) was the demographic variable that had an impact on the residents’ altruism factor. We believe that with the rapid growth of China’s economy, young people face greater pressure of social competition, which makes them pay more attention to their own interests [53]. After adding the potential explanatory variables, the variables coefficient and significance level of the baseline model were unchanged, and the adjusted R-squared increased, showing that the potential explanatory variables we selected are reliable. As shown in Table 4, the results of multiple regression analysis showed that policy (β2 = 0.029, *p* < 0.1), information intervention (β2 = 0.130, *p* < 0.01), and the knowledge of household waste separation (β2 = 0.048, *p* < 0.05) all had positive and significant effects on the altruism factor. It was also indicated that external interventions might help promote the altruism factor in residents since these factors easily form a positive social atmosphere. Based on this, the authorities can cultivate such a social atmosphere through information publicity and some related education.

The results of this study may be similar in other cities in China, as the main findings (e.g., subjective norm and PBC have positive and significant effects on household waste separation intention) are consistent with recent case studies in mainland China [41,48]. However, since European and American cities pursue individualism, which is different from collectivism in mainland China [54], the results of this study may be different.

## 6. Conclusions

The contrast between rational choice and the altruism factor is a central theme in social sciences. In this study, we explored the explanatory efficacy of two different tendencies on household waste separation behavior. The analyses were applied to a case study in Xi’an, China, a city which has a per capita GDP around the average level of the major cities in the country. Through the structural equation model test, we found that the rational choice model could better explain residents’ behavior toward household waste separation. The altruism factor cannot directly affect waste separation behavior at the source, but it may have an indirect impact on such behavior through subjective norms and PBC. Additionally, the altruism factor was highly correlated with the attitude determinant of household waste separation behavior. In summary, this study supported the view that the altruism factor should be incorporated into a rational model to enhance its explanatory efficacy. Furthermore, some factors influencing residents’ altruism factor to household waste separation were identified, including age, policy, information intervention, and knowledge. Finally, the practical implication is that the authorities can cultivate an altruistic social atmosphere through information publicity and some related education to promote residents’ behavior toward household waste separation.

## Figures and Tables

**Table 1:** The distribution of demographic characteristics and living area (*n* = 1102).

Category	Response	Frequency	Percentage (%)
Gender			
	Male	439	39.84
	Female	663	60.16
Age			
	7–14	153	13.88
	15–24	124	11.25
	25–34	204	18.51
	35–44	166	15.06
	45–54	120	10.89
	55–64	178	16.15
	Over 64	157	14.25
Education level			
	Illiterate	15	1.36
	Elementary school	162	14.70
	Junior high school	199	18.06
	Senior high school	257	23.32
	Junior college or Bachelor’s	406	36.84
	Master’s and above	63	5.72
Monthly income (RMB)			
	Below 2500	440	39.93
	2501–4000	238	21.60
	4001–6000	188	17.06
	6001–8000	100	9.07
	8001–10,000	55	4.99
	10,001–20,000	62	5.63
	Over 20,000	19	1.72
Living area			
	Baqiao	220	19.96
	Beilin	144	13.07
	Lianhu	184	16.70
	Weiyang	173	15.70
	Xincheng	165	14.97
	Yanta	216	19.60

All participants have lived in their community for more than one year.

**Table 2:** Test results of discriminant validity.

	AF	PBC	SN	AD	HWSI	HWSB
AF	0.264					
PBC	0.114	0.429				
SN	0.090	0.039	0.503			
AD	0.216	0.094	0.074	0.219		
HWSI	0.086	0.195	0.130	0.072	0.272	
HWSB	0.078	0.159	0.107	0.065	0.220	0.761

AF = altruism factor; AD = attitude determinant; SN = subject norm; PBC = perceived behavioral control; HWSI = household waste separation intention; HWSB = household waste separation behavior.

**Table 3:** Test results of research hypotheses.

Hypotheses	Relationships	Estimates	S.E.	*p*-Values
H1	AD→HWSI	0.04	0.099	ns
H2	SN→HWSI	0.29	0.024	<0.001
H3	PBC→HWSI	0.52	0.034	<0.001
H4	HWSI→HWSB	0.48	0.053	<0.001
H5	AF→HWSI	0.04	0.091	ns
H6	AF→HWSB	0.02	0.051	ns
H7	AF→AD	0.90	0.031	<0.001
H8	AF→SN	0.25	0.047	<0.001
H9	AF→PBC	0.34	0.050	<0.001

AF = altruism factor; AD = attitude determinant; SN = subject norm; PBC = perceived behavioral control; HWSI = household waste separation intention; HWSB = household waste separation behavior; S.E. means standard error; “ns” means not significant (*p* > 0.1).

**Table 4:** TPB accounted for the variance in intention and behavior.

	Without Altruism Factor	With Altruism Factor
Household waste separation intention	38%	42%
Household waste separation behavior	22%	23%

**Table 5:** Multiple regression analysis of influencing factors of altruism.

Variables	Baseline Model			Including the Additional Variables		
	β1	*t*-Statistics	*p*-Values	β2	*t*-Statistics	*p*-Values
Gender	−0.036	−0.99	ns	−0.021	−0.61	ns
Age	0.028	3.75	<0.01	0.014	1.99	<0.05
Education	−0.017	−0.96	ns	−0.016	−0.91	ns
Income	0.009	0.56	ns	0.008	0.52	ns
Policy				0.029	1.87	<0.1
Information intervention				0.130	4.72	<0.01
Knowledge				0.048	2.56	<0.05
*n*	1102			1102		
R-squared	0.012			0.070		

The model passed the multicollinearity test; “ns” means not significant (*p* > 0.1).

## Data Availability

The data are not publicly available in accordance with consent provided by participants on the use of confidential data.

## Appendix A

**Table A1:** Questionnaire of the Survey.

Constructs	Tag	Questions
Attitude determinant (AD)	AD1	Household waste separation is good.
	AD2	Household waste separation is rewarding.
	AD3	Household waste separation is hygienic.
	AD4	Household waste separation is pleasant.
Subject norm (SN)	SN1	My family expect me to separate household waste.
	SN2	My friends expect me to separate household waste.
	SN3	My neighbors expect me to separate household waste.
	SN4	The government expect me to separate household waste.
	SN5	Environmental organization expect me to separate household waste.
Perceived behavioral control (PBC)	PBC1	I know how to separate household waste.
	PBC2	Household waste separation is easy.
	PBC3	Whether or not I separate household waste is entirely up to me.
	PBC4	I have a lot of time to separate household waste.
	PBC5	I have enough space in my house to store the separated waste.
Altruism factor (AF)	AF1	Household waste separation can decrease environmental pollution for public health.
	AF2	Failure to separate household waste will damage public environment.
	AF3	Household waste separation can reduce the waste of public resources.
	AF4	Household waste separation can provide a better environment for others (such as human offspring).
Household waste separation intention (HWSI)	HWSI1	I am willing to separate household waste.
	HWSI2	I plan to separate household waste.
	HWSI3	I will try my best to separate household waste.
Household waste separation behavior (HWSB)	HWSB	I never/often separate my household waste.
